# 2-Chloro-5-methyl-3-nitro­pyridine

**DOI:** 10.1107/S160053681104400X

**Published:** 2011-10-29

**Authors:** Da-Tong Zhang, Ling-Yan Huo

**Affiliations:** a School of Chemistry and Pharmaceutical Engineering, Shandong Polytechnic University, Jinan 250353, People’s Republic of China

## Abstract

The title compound, C_6_H_5_ClN_2_O_2_, crystallizes with two independent mol­ecules in the asymmetric unit. Inter­molecular C—H⋯O hydrogen bonds stabilize the crystal structure.

## Related literature

For aplication of pyridines, see: Madsen-Duggan *et al.* (2010 ▶); Meurer *et al.* (2005 ▶); Liégeois *et al.* (1993 ▶); Kagabu *et al.* (2005 ▶). For related structures, see: Ng (2010 ▶). For standard bond lengths, see: Allen *et al.* (1987 ▶).
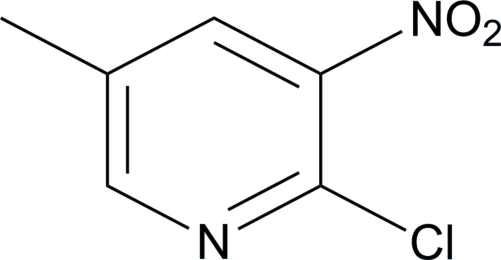


## Experimental

### 

#### Crystal data

C_6_H_5_ClN_2_O_2_
*M* *_r_* = 172.57
Orthorhombic, 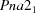

*a* = 21.435 (6) Å
*b* = 8.151 (2) Å
*c* = 8.494 (2) Å
*V* = 1484.0 (7) Å^3^
*Z* = 8
Mo *K*α radiation
μ = 0.46 mm^−1^
*T* = 298 K
0.38 × 0.24 × 0.21 mm

#### Data collection

Bruker SMART CCD area-detector diffractometer
7071 measured reflections
2134 independent reflections
1749 reflections with *I* > 2σ(*I*)
*R*_int_ = 0.023

#### Refinement

*R*[*F* ^2^ > 2σ(*F* ^2^)] = 0.037
*wR*(*F* ^2^) = 0.093
*S* = 1.06
2134 reflections
201 parameters
1 restraint
H-atom parameters constrained
Δρ_max_ = 0.20 e Å^−3^
Δρ_min_ = −0.13 e Å^−3^
Absolute structure: Flack (1983) ▶, 139 Friedel pairs
Flack parameter: −0.08 (8)

### 

Data collection: *SMART* (Bruker, 1998 ▶); cell refinement: *SAINT* (Bruker, 1999 ▶); data reduction: *SAINT*; program(s) used to solve structure: *SHELXS97* (Sheldrick, 2008 ▶); program(s) used to refine structure: *SHELXL97* (Sheldrick, 2008 ▶); molecular graphics: *SHELXTL* (Sheldrick, 2008 ▶); software used to prepare material for publication: *SHELXTL*.

## Supplementary Material

Crystal structure: contains datablock(s) global, I. DOI: 10.1107/S160053681104400X/hg5113sup1.cif

Structure factors: contains datablock(s) I. DOI: 10.1107/S160053681104400X/hg5113Isup2.hkl

Supplementary material file. DOI: 10.1107/S160053681104400X/hg5113Isup3.cml

Additional supplementary materials:  crystallographic information; 3D view; checkCIF report

## Figures and Tables

**Table 1:** Hydrogen-bond geometry (Å, °)

*D*—H⋯*A*	*D*—H	H⋯*A*	*D*⋯*A*	*D*—H⋯*A*
C9—H9*A*⋯O2^i^	0.93	2.51	3.243 (4)	136

Symmetry code: (i) 
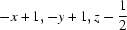
.

**Fig. 1.**
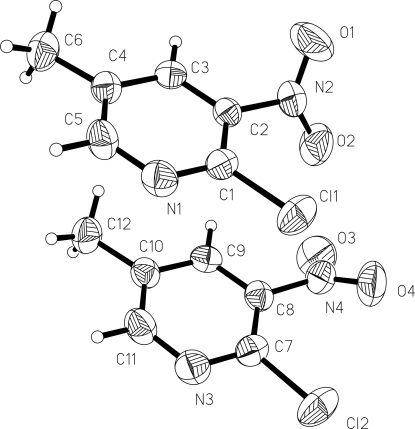
View of the title compound (I), with displacement ellipsoids drawn at the 40% probability level.

## supplementary crystallographic information

### Comment

Substituted pyridines are often used as pharmacophores in medicinal chemistry (Madsen-Duggan *et al.*, 2010; Meurer *et al.*, 2005). 2-Chloro-5-methyl-3-nitropyridine (I) is important intermediate in the synthesis of some bioactive products (Liégeois, *et al.*, 1993; Kagabu, *et al.*, 2005). The title compound was prepared by the chlorination of 2-hydroxy-5-methyl-3-nitropyridine with thionyl chloride. We present here the crystal structure of (I).

The title compound, C_6_H_5_ClN_2_O_2_, crystallizes with two independent molecules in the asymmetric unit. All bond lengths in the molecular are normal (Allen *et al.*, 1987) and in a good agreement with those reported previously (Ng, 2010). C—H···O intermolecular hydrogen bonds stabilize the crystal structure.(Table 1).

### Experimental

The title compound was synthesized by the reaction of 2-hydroxy-5-methyl-3-nitropyridine (0.01 mol) with thionyl chloride (15 ml) in the presence of a small amount of DMF at reflux (3 h). After evaporation, the reaction residue was diluted with water. The aqueous solution was extracted with dichloromethane, and the organic phase was dried and evaporated to afford the title product in 92% isolated yield. Crystals suitable for X-ray diffraction analysis were obtained by slow evaporation of a solution of the title compound in a hexane/methylene chloride mixture (1:1 *v*/*v*) at room temperature over a period of one week.

### Refinement

All H atoms were found on difference maps, with C—H = 0.93–0.96 Å and included in the final cycles of refinement using a riding model, with *U*_iso_(H) = 1.2*U*_eq_(C) and 1.5*U*_eq_(C) for the methyl H atoms.

### Crystal data

**Table:**

C_6_H_5_ClN_2_O_2_	*F*(000) = 704
*M**_r_* = 172.57	*D*_x_ = 1.545 Mg m^−^^3^
Orthorhombic, *P**n**a*2_1_	Mo *K*α radiation, λ = 0.71073 Å
Hall symbol: P 2c -2n	Cell parameters from 1564 reflections
*a* = 21.435 (6) Å	θ = 2.3–22.0°
*b* = 8.151 (2) Å	µ = 0.46 mm^−^^1^
*c* = 8.494 (2) Å	*T* = 298 K
*V* = 1484.0 (7) Å^3^	Block, colorless
*Z* = 8	0.38 × 0.24 × 0.21 mm

### Data collection

**Table:**

Bruker SMART CCD area-detector diffractometer	1749 reflections with *I* > 2σ(*I*)
Radiation source: fine-focus sealed tube	*R*_int_ = 0.023
graphite	θ_max_ = 25.0°, θ_min_ = 1.9°
φ and ω scans	*h* = −25→25
7071 measured reflections	*k* = −9→9
2134 independent reflections	*l* = −10→6

### Refinement

**Table:**

Refinement on *F*^2^	Secondary atom site location: difference Fourier map
Least-squares matrix: full	Hydrogen site location: inferred from neighbouring sites
*R*[*F*^2^ > 2σ(*F*^2^)] = 0.037	H-atom parameters constrained
*wR*(*F*^2^) = 0.093	*w* = 1/[σ^2^(*F*_o_^2^) + (0.0538*P*)^2^] where *P* = (*F*_o_^2^ + 2*F*_c_^2^)/3
*S* = 1.06	(Δ/σ)_max_ = 0.001
2134 reflections	Δρ_max_ = 0.20 e Å^−^^3^
201 parameters	Δρ_min_ = −0.13 e Å^−^^3^
1 restraint	Absolute structure: Flack (1983), 139 Friedel pairs
Primary atom site location: structure-invariant direct methods	Flack parameter: −0.08 (8)

### Special details

**Table:**

Geometry. All e.s.d.'s (except the e.s.d. in the dihedral angle between two l.s. planes) are estimated using the full covariance matrix. The cell e.s.d.'s are taken into account individually in the estimation of e.s.d.'s in distances, angles and torsion angles; correlations between e.s.d.'s in cell parameters are only used when they are defined by crystal symmetry. An approximate (isotropic) treatment of cell e.s.d.'s is used for estimating e.s.d.'s involving l.s. planes.
Refinement. Refinement of *F*^2^ against ALL reflections. The weighted *R*-factor *wR* and goodness of fit *S* are based on *F*^2^, conventional *R*-factors *R* are based on *F*, with *F* set to zero for negative *F*^2^. The threshold expression of *F*^2^ > σ(*F*^2^) is used only for calculating *R*-factors(gt) *etc*. and is not relevant to the choice of reflections for refinement. *R*-factors based on *F*^2^ are statistically about twice as large as those based on *F*, and *R*- factors based on ALL data will be even larger.

### Fractional atomic coordinates and isotropic or equivalent isotropic displacement parameters (Å^2^)

**Table:**

	*x*	*y*	*z*	*U*_iso_*/*U*_eq_	
Cl1	0.64412 (4)	0.34108 (9)	0.69639 (8)	0.0705 (3)	
Cl2	0.64313 (4)	0.85811 (9)	0.70504 (10)	0.0812 (3)	
O1	0.50283 (10)	0.0831 (3)	0.5349 (5)	0.1076 (10)	
O2	0.51384 (10)	0.3414 (3)	0.5424 (4)	0.0821 (7)	
O3	0.48112 (10)	0.7214 (3)	0.4606 (4)	0.0982 (9)	
O4	0.52431 (11)	0.6869 (3)	0.6857 (4)	0.0952 (8)	
N1	0.70207 (10)	0.2297 (3)	0.4520 (3)	0.0583 (6)	
N2	0.53214 (10)	0.2046 (3)	0.5159 (3)	0.0572 (6)	
N3	0.69533 (11)	0.7387 (3)	0.4591 (3)	0.0640 (6)	
N4	0.52614 (12)	0.7020 (3)	0.5437 (4)	0.0652 (7)	
C1	0.64679 (12)	0.2463 (3)	0.5147 (3)	0.0462 (6)	
C2	0.59406 (10)	0.1857 (3)	0.4440 (3)	0.0404 (6)	
C3	0.59813 (12)	0.1067 (3)	0.3021 (3)	0.0443 (6)	
H3B	0.5626	0.0632	0.2549	0.053*	
C4	0.65528 (11)	0.0926 (3)	0.2303 (3)	0.0467 (7)	
C5	0.70548 (13)	0.1550 (3)	0.3124 (4)	0.0591 (7)	
H5A	0.7447	0.1443	0.2670	0.071*	
C6	0.66285 (14)	0.0156 (4)	0.0719 (4)	0.0673 (8)	
H6A	0.6313	−0.0665	0.0572	0.101*	
H6B	0.7033	−0.0343	0.0647	0.101*	
H6C	0.6588	0.0982	−0.0081	0.101*	
C7	0.64127 (13)	0.7526 (3)	0.5297 (3)	0.0513 (7)	
C8	0.58714 (12)	0.6920 (3)	0.4649 (3)	0.0479 (7)	
C9	0.58893 (12)	0.6180 (3)	0.3199 (3)	0.0482 (7)	
H9A	0.5525	0.5788	0.2740	0.058*	
C10	0.64506 (11)	0.6021 (3)	0.2429 (3)	0.0500 (8)	
C11	0.69626 (13)	0.6619 (4)	0.3200 (4)	0.0634 (8)	
H11A	0.7349	0.6480	0.2717	0.076*	
C12	0.65035 (14)	0.5240 (4)	0.0832 (4)	0.0687 (8)	
H12A	0.6920	0.4835	0.0684	0.103*	
H12B	0.6411	0.6040	0.0035	0.103*	
H12C	0.6213	0.4347	0.0755	0.103*	

### Atomic displacement parameters (Å^2^)

**Table:**

	*U*^11^	*U*^22^	*U*^33^	*U*^12^	*U*^13^	*U*^23^
Cl1	0.0895 (6)	0.0727 (5)	0.0492 (5)	0.0039 (4)	−0.0117 (4)	−0.0149 (5)
Cl2	0.1138 (7)	0.0757 (6)	0.0542 (6)	0.0059 (4)	−0.0185 (5)	−0.0151 (6)
O1	0.0742 (14)	0.0879 (17)	0.161 (3)	−0.0239 (14)	0.0468 (16)	0.006 (2)
O2	0.0775 (14)	0.0813 (15)	0.0875 (18)	0.0228 (11)	0.0239 (14)	−0.0082 (13)
O3	0.0574 (14)	0.118 (2)	0.119 (3)	0.0116 (14)	−0.0001 (14)	−0.0181 (17)
O4	0.1076 (19)	0.1013 (18)	0.0766 (19)	0.0100 (14)	0.0405 (16)	−0.0110 (17)
N1	0.0416 (12)	0.0721 (15)	0.0613 (17)	−0.0080 (11)	−0.0047 (12)	−0.0035 (13)
N2	0.0503 (13)	0.0667 (16)	0.0547 (16)	0.0012 (12)	0.0115 (12)	0.0037 (13)
N3	0.0563 (14)	0.0801 (16)	0.0555 (16)	−0.0106 (12)	−0.0083 (14)	−0.0021 (14)
N4	0.0648 (18)	0.0604 (16)	0.071 (2)	0.0057 (13)	0.0145 (16)	−0.0095 (15)
C1	0.0549 (15)	0.0421 (13)	0.0415 (16)	0.0006 (11)	−0.0035 (13)	0.0015 (12)
C2	0.0403 (13)	0.0406 (13)	0.0404 (16)	0.0003 (10)	0.0005 (11)	0.0043 (11)
C3	0.0434 (14)	0.0413 (13)	0.0483 (16)	−0.0016 (10)	−0.0047 (13)	0.0013 (12)
C4	0.0477 (14)	0.0492 (13)	0.0433 (19)	0.0028 (11)	0.0002 (13)	0.0040 (12)
C5	0.0425 (14)	0.0787 (19)	0.0563 (19)	0.0001 (13)	0.0060 (14)	0.0006 (17)
C6	0.0776 (18)	0.074 (2)	0.0503 (18)	0.0075 (16)	0.0082 (16)	−0.0046 (16)
C7	0.0661 (17)	0.0473 (15)	0.0406 (16)	0.0024 (12)	−0.0097 (14)	0.0052 (12)
C8	0.0515 (15)	0.0444 (14)	0.0479 (17)	0.0034 (11)	0.0037 (13)	0.0076 (13)
C9	0.0498 (15)	0.0443 (13)	0.0505 (18)	−0.0049 (11)	−0.0076 (13)	0.0049 (13)
C10	0.0553 (17)	0.0498 (15)	0.0449 (19)	−0.0011 (12)	0.0034 (13)	0.0076 (13)
C11	0.0455 (15)	0.084 (2)	0.061 (2)	−0.0082 (14)	0.0031 (15)	0.0033 (18)
C12	0.0832 (19)	0.076 (2)	0.0468 (18)	−0.0011 (17)	0.0098 (16)	−0.0015 (16)

### Geometric parameters (Å, °)

**Table:**

Cl1—C1	1.727 (3)	C4—C5	1.380 (4)
Cl2—C7	1.720 (3)	C4—C6	1.494 (4)
O1—N2	1.184 (3)	C5—H5A	0.9300
O2—N2	1.203 (3)	C6—H6A	0.9600
O3—N4	1.206 (4)	C6—H6B	0.9600
O4—N4	1.213 (4)	C6—H6C	0.9600
N1—C1	1.306 (3)	C7—C8	1.376 (4)
N1—C5	1.335 (4)	C8—C9	1.372 (4)
N2—C2	1.469 (3)	C9—C10	1.376 (4)
N3—C7	1.310 (4)	C9—H9A	0.9300
N3—C11	1.338 (4)	C10—C11	1.368 (4)
N4—C8	1.471 (4)	C10—C12	1.502 (4)
C1—C2	1.372 (3)	C11—H11A	0.9300
C2—C3	1.369 (4)	C12—H12A	0.9600
C3—C4	1.373 (4)	C12—H12B	0.9600
C3—H3B	0.9300	C12—H12C	0.9600
			
C1—N1—C5	117.3 (2)	H6A—C6—H6B	109.5
O1—N2—O2	125.2 (3)	C4—C6—H6C	109.5
O1—N2—C2	116.6 (3)	H6A—C6—H6C	109.5
O2—N2—C2	118.0 (2)	H6B—C6—H6C	109.5
C7—N3—C11	117.3 (2)	N3—C7—C8	122.1 (3)
O3—N4—O4	124.7 (3)	N3—C7—Cl2	114.8 (2)
O3—N4—C8	116.9 (3)	C8—C7—Cl2	123.1 (2)
O4—N4—C8	118.4 (3)	C9—C8—C7	119.6 (3)
N1—C1—C2	122.1 (3)	C9—C8—N4	117.2 (3)
N1—C1—Cl1	116.2 (2)	C7—C8—N4	123.2 (3)
C2—C1—Cl1	121.7 (2)	C8—C9—C10	119.5 (3)
C3—C2—C1	120.1 (2)	C8—C9—H9A	120.2
C3—C2—N2	118.2 (2)	C10—C9—H9A	120.2
C1—C2—N2	121.6 (2)	C11—C10—C9	116.1 (3)
C2—C3—C4	119.2 (2)	C11—C10—C12	121.5 (3)
C2—C3—H3B	120.4	C9—C10—C12	122.4 (3)
C4—C3—H3B	120.4	N3—C11—C10	125.3 (3)
C3—C4—C5	116.1 (3)	N3—C11—H11A	117.3
C3—C4—C6	122.2 (3)	C10—C11—H11A	117.3
C5—C4—C6	121.7 (3)	C10—C12—H12A	109.5
N1—C5—C4	125.1 (3)	C10—C12—H12B	109.5
N1—C5—H5A	117.5	H12A—C12—H12B	109.5
C4—C5—H5A	117.5	C10—C12—H12C	109.5
C4—C6—H6A	109.5	H12A—C12—H12C	109.5
C4—C6—H6B	109.5	H12B—C12—H12C	109.5
			
C5—N1—C1—C2	2.0 (4)	C11—N3—C7—C8	−0.2 (4)
C5—N1—C1—Cl1	179.2 (2)	C11—N3—C7—Cl2	−178.1 (2)
N1—C1—C2—C3	−0.9 (4)	N3—C7—C8—C9	−1.7 (4)
Cl1—C1—C2—C3	−177.95 (19)	Cl2—C7—C8—C9	176.00 (19)
N1—C1—C2—N2	179.7 (2)	N3—C7—C8—N4	178.3 (3)
Cl1—C1—C2—N2	2.7 (3)	Cl2—C7—C8—N4	−4.0 (4)
O1—N2—C2—C3	55.0 (4)	O3—N4—C8—C9	−34.7 (4)
O2—N2—C2—C3	−120.8 (3)	O4—N4—C8—C9	143.8 (3)
O1—N2—C2—C1	−125.7 (3)	O3—N4—C8—C7	145.3 (3)
O2—N2—C2—C1	58.5 (4)	O4—N4—C8—C7	−36.2 (4)
C1—C2—C3—C4	−1.5 (4)	C7—C8—C9—C10	1.5 (4)
N2—C2—C3—C4	177.8 (2)	N4—C8—C9—C10	−178.5 (2)
C2—C3—C4—C5	2.6 (4)	C8—C9—C10—C11	0.6 (4)
C2—C3—C4—C6	−176.9 (2)	C8—C9—C10—C12	−179.3 (2)
C1—N1—C5—C4	−0.8 (4)	C7—N3—C11—C10	2.5 (5)
C3—C4—C5—N1	−1.6 (4)	C9—C10—C11—N3	−2.7 (5)
C6—C4—C5—N1	177.9 (3)	C12—C10—C11—N3	177.2 (3)

### Hydrogen-bond geometry (Å, °)

**Table:**

*D*—H···*A*	*D*—H	H···*A*	*D*···*A*	*D*—H···*A*
C9—H9A···O2^i^	0.93	2.51	3.243 (4)	136.

Symmetry codes: (i) −*x*+1, −*y*+1, *z*−1/2.
